# Association of monocyte myeloperoxidase with incident cardiovascular disease: The Atherosclerosis Risk in Communities Study

**DOI:** 10.1371/journal.pone.0205310

**Published:** 2018-10-09

**Authors:** Abayomi O. Oyenuga, David Couper, Kunihiro Matsushita, Eric Boerwinkle, Aaron R. Folsom

**Affiliations:** 1 Division of Epidemiology and Community Health, School of Public Health, University of Minnesota, Minneapolis, Minnesota, United States of America; 2 Department of Biostatistics, Gillings School of Global Public Health, The University of North Carolina at Chapel Hill, Chapel Hill, North Carolina, United States of America; 3 Department of Epidemiology, Welch Center for Prevention, Epidemiology, and Clinical Research, Johns Hopkins Bloomberg School of Public Health, Baltimore, Maryland, United States of America; 4 Department of Epidemiology, Human Genetics and Environmental Sciences, School of Public Health, The University of Texas Health Science Center at Houston, Houston, Texas, United States of America; Medizinische Universitat Innsbruck, AUSTRIA

## Abstract

Myeloperoxidase (MPO) is a heme-containing peroxidase found in azurophilic granules of neutrophils and monocytes. Epidemiological studies have reported greater plasma MPO concentration to be associated with increased incidence of several cardiovascular diseases (CVD), but the association of intracellular monocyte MPO (mMPO) with CVD is unclear. The prospective population-based Atherosclerosis Risk in Communities (ARIC) cohort study measured mMPO using flow cytometry in 1,465 participants. The association of mMPO with incident cardiovascular disease (CVD, comprising incident coronary heart disease (CHD), heart failure, stroke, peripheral artery disease, and cardiovascular mortality) was examined over a median 9.6 years of follow-up (n = 290 CVD events). There was no statistically significant association between mMPO and all incident CVD events in either age, sex, and race-adjusted proportional hazards models (HR (95% CI) across tertiles of mMPO: 1, 1.09 (0.76, 1.57), and 0.78 (0.52, 1.15), P-trend = 0.21) or adjusted for other major CVD risk factors (HR (95% CI): 1, 1.17 (0.81, 1.69), and 0.87 (0.58, 1.29), P-trend = 0.50). There also was no association between mMPO tertiles and incident CHD, heart failure, or all-cause mortality, examined separately. In conclusion, intracellular monocyte myeloperoxidase was not associated with incident cardiovascular disease in this prospective population-based study.

## Introduction

Myeloperoxidase (MPO) is a heme-containing peroxidase expressed largely by cells of the myeloid lineage [1,2]. It is found mostly in azurophilic granules of neutrophils and monocytes, and to some extent in Kupffer cells, microglia, granule-containing neurons, and pyramidal neurons of the hippocampus [1]. It is released into extracellular fluids during its synthesis and in the setting of inflammation [3,4]. Most of the actions of MPO stem from its catalytic activities on the reaction between hydrogen peroxide and halides. The resulting products of these reactions are HOCl and other hypohalites. It is also involved in the oxidation of plasma lipoproteins, cross-linking of proteins and generation of other highly reactive molecules, such as tyrosyl radicals [1,2]. While the products of these reactions are important in the body’s response to microbial agents, recent evidence suggests that hypohalous acid reactions with lipoproteins, nitric oxide synthase, and endothelial cells promote atherosclerosis and other vascular diseases [5]. Several epidemiological studies have shown a positive association between concentrations of plasma MPO and cardiovascular disease (CVD) [6–9].

Several inflammatory cell types are involved in the pathogenesis of atherosclerosis, and consequently, its cardiovascular disease (CVD) outcomes [10]. Of these cells, monocytes/macrophages are the most important [11]. Monocytes/macrophages have a longer half-life compared to neutrophils [12,13] and contribute more to CVD outcomes compared to other MPO-positive cells [11,14]. Transmigration of monocytes from circulation into tissue, important in the body’s inflammatory response to pathogens, is highly relevant to the initiation and progression of atherosclerosis. Several cell signals are employed in the facilitation of this process [10], but recently it has been demonstrated that MPO is involved in the electrostatic signaling that enhances monocyte adhesion and transmigration [2]. Considering this, it is possible that reduced secretion of MPO, and therefore higher intracellular MPO, is associated with lower MPO-related tissue transmigration and consequently lower incidence of atherosclerosis.

MPO is synthesized during myeloid differentiation in bone marrow, and only promyelocytes, promyelomonocytes, and monocyte precursors actively synthesize MPO. Circulating monocytes do not actively synthesize MPO. Given the varied origins of plasma MPO [1,2] intracellular MPO might provide more accurate information on the potential for and degree of tissue-specific activities of MPO-positive inflammatory cells [15,16]. Since circulating monocytes are more accessible than resident tissue macrophages, a better understanding of the association between intracellular monocyte MPO and CVD might help inform better strategies for risk modification for CVD [14,16].

Epidemiological studies have scarcely examined whether there is an association of myeloperoxidase within monocytes/macrophages with incident CVD. In a cross-sectional study, the Atherosclerosis Risk in Communities (ARIC) Study found monocyte MPO (mMPO) to be negatively, not positively, associated with the prevalence of peripheral artery disease (PAD) [17]. This negative relation was believed to be explained by release and depletion of mMPO during monocyte activation in PAD patients [17]. With 10 years of follow-up since MPO measurement, we studied the association of monocyte myeloperoxidase and incident CVD in ARIC.

## Materials and methods

### Study population

The ARIC Study (S1 Table) involves a prospective community-based cohort study to identify risk factors for atherosclerosis and CVD. Participants were recruited between 1987 and 1989 from 4 communities in the United States (Washington County, Maryland; suburban Minneapolis, Minnesota; Jackson, Mississippi; and Forsyth County, North Carolina) [18]. Participants subsequently have been examined at periodic study visits. Participants are also followed up by annual or semiannual telephone interviews. The institutional review boards of the Universities of Minnesota, Mississippi, North Carolina, and Texas, as well as Johns Hopkins, Baylor, and Wake Forest Universities approved the ARIC protocol. All participants provided written informed consent.

In 2005–2006 a stratified sample of surviving participants were re-examined as part of an ARIC ancillary study (the ARIC Carotid MRI study) [19]. The sampling goal was to recruit 1200 participants with high values of maximum carotid artery wall thickness and 800 individuals randomly sampled from the remainder of the carotid artery wall thickness distribution. Potential recruits with safety concerns for the MRI examination were excluded, and the final sample examined was 2,066. The full details of the selection process have been published in previous reports [19,20].

For this report, of those 2,066 in the ARIC Carotid MRI study, we excluded 601 individuals who had missing mMPO data (n = 141), missing covariates (n = 45), or a history of PAD, myocardial infarction, or heart failure (n = 415), leaving final study sample of 1,465 participants (830 from the high IMT group and 635 from the random sample).

### mMPO measurement by flow cytometry

The methods for blood sampling and flow cytometry have been described in detail in previous ARIC publications [17,21]. Briefly, fasting blood samples were collected at the ARIC Carotid MRI examination in Cyto-Chex BCT vacutainer tubes (Streck, Omaha, Nebraska) containing EDTA and a cell membrane stabilizer for blood cells. They were shipped to the ARIC flow cytometry laboratory by overnight courier, and samples were analyzed immediately upon arrival. Monocyte myeloperoxidase was measured using whole blood flow cytometry (Coulter Epics XL, Beckman Coulter, Inc., Miami, FL). Of the markers measured, the primary variable of interest for this analysis was intracellular monocyte MPO expressed as the median fluorescence intensity (MFI), because of ARIC’s previous cross-sectional report on an inverse association of mMPO with PAD [17].

### Covariates

Covariates used in the analysis were measured at the Carotid MRI exam. Participants reported their race, use of antihypertensive medication within the previous two weeks, and use of cholesterol-lowering medication. Cigarette-smoking status was categorized as current, former, or never. Seated, resting blood pressure was measured using a random-zero sphygmomanometer; the average of the last 2 out of 3 readings was used. Body mass index (BMI; kg/m^2^) was calculated using measured weight and height. Diabetes mellitus was defined as fasting blood glucose ≥ 126 mg/dl (7.0 mmol/L), non-fasting glucose ≥ 200 mg/dl (11.1 mmol/L), treatment for diabetes mellitus, or self-reported physician diagnosis of diabetes mellitus. Total cholesterol, triglycerides, and HDL-cholesterol were assayed from plasma samples as described previously [22].

### Endpoint assessment

Incident CVD events occurring between date of the ARIC Carotid MRI examination in 2005–6 and December 31, 2015 were identified, blinded to exposure, through: 1) annual telephone calls to cohort participants (or proxy); 2) active surveillance of local hospital discharge indexes; 3) searches of state death records; and 4) linkage to the National Death Index. Trained abstractors collected information on all hospitalizations involving cardiovascular disease. Coronary heart disease (CHD) events were defined as a definite or probable myocardial infarction or definite fatal CHD by physician review using ARIC criteria [23] or as a coronary revascularization discharge code. For stroke classification, signs, symptoms, neuroimaging, and other diagnostic reports were used in a computer algorithm and by physician reviewers using criteria adapted from the National Survey of Stroke [24,25]. Ischemic stroke was defined as a validated definite or probable embolic or thrombotic brain infarction. Incident peripheral artery disease (PAD) was defined by hospital discharge diagnosis with International Classification of Disease, Ninth Revision (ICD-9) codes consistent with PAD, leg amputation, or leg revascularization procedures (leg endarterectomy, aorto-iliac-femoral bypass surgery, or other leg bypass or angioplastic surgery). Heart failure (HF) was defined by a hospitalization with an ICD 9 code of 428 or death with an underlying cause of ICD-9 code 428 or ICD-10 code I50 [26]. Total CVD mortality was defined as deaths with underlying causes of ICD‐9 codes 390‐459 or ICD‐10 codes I00‐I99.

We also created a composite endpoint for all incident cardiovascular events. This variable comprised incident CHD, incident ischemic stroke, incident PAD, incident heart failure, and CVD deaths. Follow-up ended at the date of first event, loss to follow-up, death, or else December 31, 2015.

### Statistical analysis

Our hypothesis was that mMPO would be inversely associated with incident CVD outcomes. The main statistical analyses were performed using SAS 9.4 (SAS Institute Inc., Cary, NC). To account for the complex survey design of the ARIC Carotid MRI study, our analyses were weighted by the inverse probabilities of sampling each stratum; analyses were done using the appropriate SAS *weight* statements, *strata* statements, and survey analysis procedures without finite population correction. Relations between mMPO Tertiles and the covariates were examined as weighted means and proportions for continuous and categorical variables, respectively. Multivariable Cox regression models were used to compute hazard ratios relating mMPO Tertiles to incident CVD, after sample weighting and adjustment for age, sex, and race in Model 1, and additionally for major CVD risk factors in Model 2. The crude incidence rates were computed using Poisson regression. Four adverse outcomes were examined: all CVD events (the composite outcome), heart failure, CHD, and all-cause mortality. There were too few events to study incident PAD, stroke, or CVD deaths separately. We tested for linear trends in the relationships of the covariates and mMPO and the associations of the CVD outcome variables and mMPO tertiles by using an ordinal variable incorporating the tertile number.

## Results

The baseline (2005–2006) age range for the 1,465 ARIC participants at risk of CVD was 60–84 years (mean = 70). Approximately 58% were women, and 21% were black. The median mMPO MFI was 90.6. Table 1 shows the weighted demographic and risk factor characteristics by mMPO tertiles. A greater proportion of women and blacks and a smaller proportion of current cigarette smokers were in the higher mMPO tertiles; mMPO was associated positively with BMI and negatively with age. There was no statistically significant association between mMPO and diabetes, systolic blood pressure, use of antihypertensive medication, total cholesterol, HDL-cholesterol, triglycerides, or use of cholesterol-lowering medication.

**Table 1:** Participant characteristics according to plasma monocyte myeloperoxidase median fluorescence intensity (MFI) tertiles, ARIC, 2005–2006.

Characteristics*	Monocyte Myeloperoxidase MFI Tertiles			
	Tertile 1 (6.1–79.9)	Tertile 2 (80.6–100.0)	Tertile 3 (100.9–209.1)	P-trend
N	484	496	485	
Age (years)	70.7 (0.3)	69.8 (0.3)	69.5 (0.3)	0.006
Sex (% female)	50.0	58.2	65.2	<0.0001
Race (% black)	14.4	18.3	29.9	<0.0001
Diabetes (%)	22.0	21.2	23.8	0.56
Current cigarette smoking (%)	10.9	7.3	5.1	0.0001
Systolic blood pressure (mmHg)	126.2 (1.1)	125.3 (1.0)	126.9 (1.0)	0.61
Diastolic blood pressure (mmHg)	66.9 (0.6)	67.0 (0.5)	68.3 (0.6)	0.08
Antihypertensive medication (%)	57.7	62.2	60.8	0.41
Use of cholesterol medication (%)	42.3	37.2	37.9	0.48
Total cholesterol (mg/dl)	195.2 (2.1)	194.9 (2.3)	199.1 (2.2)	0.21
HDL-cholesterol (mg/dl)	50.2 (0.9)	50.9 (0.9)	51.3 (0.8)	0.37
Triglycerides (mg/dl)†	136 (99.3–184.3)	127.5 (92.5–173)	124.9 (92.3–178.8)	0.51
Body mass index (kg/m^2^)	28.4 (0.3)	28.8 (0.3)	29.4 (0.3)	0.02

ARIC, Atherosclerosis Risk in Communities; HDL, high density lipoprotein; MFI, median fluorescence intensity; N, number.

* Values are mean (standard error of mean) for continuous variables and percentages for categorical variables unless otherwise specified.

† Values are expressed as median (25th– 75th percentile).

### Composite CVD outcome

Over a median follow-up time of 9.6 years (maximum 10.7 years), from 2005 to 2015, there were a total of 290 incident CVD events (first episode of either PAD, ischemic stroke, CHD, heart failure, or CVD death). As shown in Table 2, the crude incident rate for all CVD events was lowest in mMPO tertile 3 (14.2 events per 1000 person-years) compared with approximately 20 events per 1000 person-years in tertiles 1 and 2. Although the rates appear lower in tertile 3 compared to tertiles 1 and 2, we found no statistically significant association between mMPO and all incident CVD events; adjusted for age, race, and sex (Model 1), the HR (95% CI) was 0.78 (0.52, 1.15) for participants with mMPO in tertile 3 compared to individuals in tertile 1. The association shown in Table 2 was attenuated and was still not statistically significant after further adjustment for other CVD risk factors in Model 2: HR (95% CI) for tertile 3 vs tertile 1 = 0.87 (0.58,1.29).

**Table 2:** Crude incidence rate and adjusted hazard ratios (HR) with 95% confidence intervals (95% CI) of all incident cardiovascular events in relation to monocyte myeloperoxidase median fluorescence intensity (MFI) tertiles, ARIC, 2005–2015.

	Monocyte Myeloperoxidase MFI Tertiles			
All incident events*	Tertile 1	Tertile 2	Tertile 3	P-trend
Events, N	109	99	82	
Person-years	4,022	4,242	4,161	
Incidence rate†	19.7	19.5	14.2	
Model 1‡ HR (95% CI)	Ref.	1.09 (0.76, 1.57)	0.78 (0.52, 1.15)	0.21
Model 2§ HR (95% CI)	Ref.	1.17 (0.81, 1.69)	0.87 (0.58,1.29)	0.50

ARIC, Atherosclerosis Risk in Communities; BMI, body mass index; CHD, coronary heart disease; CI, confidence

intervals; CVD, cardiovascular disease; HDL, high density lipoprotein; HR, hazard ratios; MFI, median fluorescence intensity; N, number; PAD, peripheral artery disease.

* All incident events: This is a composite variable consisting of incident CHD, incident stroke, incident PAD, incident heart failure, and CVD deaths occurring during the follow-up period.

† Weighted crude incidence per 1000 person-years.

‡ Model 1: adjusted for race (white, black), gender (female, male), and age (continuous).

§ Model 2: additionally adjusted for use of cholesterol lowering medication (yes, no, unknown), diabetes mellitus (yes, no, unknown), use of antihypertensive medication (yes, no, unknown), systolic blood pressure (continuous), diastolic blood pressure (continuous), BMI (continuous), total cholesterol (continuous), HDL-c (continuous), smoking status (current, former, never), pack-years of smoking (continuous), and triglycerides (continuous).

### Individual outcomes

There were 163 incident heart failure events during the follow-up period (Table 3). In model 1, participants with mMPO in tertile 3 did not have a significantly lower risk of heart failure compared to individuals in Tertile 1: HR (95% CI) = 0.68 (0.39, 1.18) (p-trend 0.16). The heart failure HR for tertile 3 versus tertile 1 of mMPO was attenuated and was still not statistically significant after further adjustment for CVD risk factors: 0.74 (0.42,1.29) (P-trend 0.29). There was no association between mMPO tertiles and incident CHD or all-cause mortality (data not shown).

**Table 3:** Crude incidence rate and adjusted hazard ratios (HR) with 95% confidence intervals (95% CI) of incident heart failure in relation to monocyte myeloperoxidase median fluorescence intensity (MFI) tertiles, ARIC, 2005–2015.

	Monocyte Myeloperoxidase MFI Tertiles			
Incident heart failure	Tertile 1	Tertile 2	Tertile 3	P-trend
Events, N	64	60	39	
Person-years	4,127	4,379	4,355	
Incidence rate*	10.3	10.2	6.9	
Model 1† HR (95% CI)	ref	1.04 (0.63,1.72)	0.68 (0.39,1.18)	0.16
Model 2‡ HR (95% CI)	ref	1.02 (0.63,1.66)	0.74 (0.42,1.29)	0.29

ARIC, Atherosclerosis Risk in Communities; BMI, body mass index; CI, confidence intervals; HDL, high density lipoprotein; HR, hazard ratios; MFI, median fluorescence intensity; N, number.

† Model 1: adjusted for race (white, black), gender (female, male), and age (continuous).

‡ Model 2: additionally adjusted for use of cholesterol lowering medication (yes, no, unknown), diabetes mellitus (yes, no, unknown), use of antihypertensive medication (yes, no, unknown), systolic blood pressure (continuous), diastolic blood pressure (continuous), BMI (continuous), total cholesterol (continuous), HDL-c (continuous), smoking status (current, former, never), pack-years of smoking (continuous), and triglycerides (continuous).

* Weighted crude incidence per 1000 person-years.

## Discussion

In this prospective population-based study, we found that a lower level of intracellular mMPO was not associated with an increased risk of CVD. Although not significant, our results suggested an inverse relationship between mMPO and incident CVD, as shown in a previous cross-sectional study for prevalent PAD in ARIC [17]. Although there is well-documented evidence for a positive association between plasma MPO and incident CVD [7–9] and an inverse association between neutrophil MPO and CHD [27], we were unable to find any studies that have explored the relationship of intracellular monocyte MPO and incident CVD. Therefore, it is difficult to compare our findings to previous work.

The studies showing a positive association between plasma MPO and CVD may not reflect the pattern for monocyte MPO and CVD [6]. Higher plasma MPO may, in fact, reflect lower levels of intracellular mMPO due to ongoing monocyte activation and release of intracellular myeloperoxidase [4]. Unfortunately, we had no concurrent measurement of plasma MPO to assess the correlation between plasma MPO levels and intracellular mMPO; thus, we are unable to validate the hypothesis that lower intracellular mMPO was due to monocyte activation and subsequent depletion of intracellular MPO. Furthermore, neutrophils contribute a larger proportion to the overall circulating MPO than do monocytes [1,17,28], therefore it is difficult to accurately estimate the contributions of secreted mMPO to the pathogenesis of incident CVD.

Single nucleotide polymorphisms that affect MPO synthesis, and consequently the intra and extracellular concentrations of MPO, have been identified [5,29]. It is likely that in our sample mMPO levels reflect differences in synthetic activity related to these polymorphisms that regulate the transcriptional activity of myeloperoxidase in the bone marrow. However, epidemiologic studies, including several meta-analyses, done to assess the association between MPO polymorphisms and cardiovascular disease have yielded inconsistent results [30–37].

There are some limitations to consider in our study. Firstly, the complex sampling design of our study might limit its generalizability. Secondly, we had a single measure of mMPO, and changes in concentrations over time would likely obscure associations with CVD, ARIC already shows just slight to moderate reliability for repeated measurements of intracellular MPO [21]. Thirdly, the power of our study to detect significant HRs for Tertile 3 versus Tertile 1 was limited by the sample size and imprecision related to the stratified sampling design. Finally, we had a limited number of incident PAD and ischemic stroke events; therefore, we could not assess the association of mMPO and these outcomes individually.

There are some strengths to our study. Our study sample was drawn from an ongoing population-based cohort. This allowed us to assess and validate incident events and ensure that the mMPO assessment occurred before the onset of clinical CVD.

In conclusion, intracellular monocyte myeloperoxidase was not evidently associated with incident cardiovascular disease in this prospective population-based study.

## Supporting information

S1 Table Strobe checklist. (PDF)

S1 File This is the SAS Code. (SAS)

S2 File This is a SAS data file. (SAS7BDAT)

S3 File This is a SAS data file. (SAS7BDAT)
