# Supplementary material for: Association of monocyte myeloperoxidase with incident cardiovascular disease: The Atherosclerosis Risk in Communities Study
Source: PLoS One. 2018 Oct 9;13(10):e0205310. doi: 10.1371/journal.pone.0205310 (PMC6177167; doi:10.1371/journal.pone.0205310)
Supplement: S1 Table — (PDF) [file pone.0205310.s001.pdf]

STROBE Statement—Checklist of items that should be included in reports of ***cohort studies***

Editor, the manuscript line numbers for each item is indicated in red.

	Item No	Recommendation
**Title and abstract** 24	1	(*a*) Indicate the study's design with a commonly used term in the title or the abstract
23-35		(*b*) Provide in the abstract an informative and balanced summary of what was done and what was found
**Introduction**		
Background/rationale 39-79	2	Explain the scientific background and rationale for the investigation being reported
Objectives 77-79	3	State specific objectives, including any prespecified hypotheses
**Methods**		
Study design 83-98	4	Present key elements of study design early in the paper
Setting 83-98	5	Describe the setting, locations, and relevant dates, including periods of recruitment, exposure, follow-up, and data collection
Participants 83-98 129-133	6	(*a*) Give the eligibility criteria, and the sources and methods of selection of participants. Describe methods of follow-up
NA		(*b*) For matched studies, give matching criteria and number of exposed and unexposed
Variables 105-126 133-150	7	Clearly define all outcomes, exposures, predictors, potential confounders, and effect modifiers. Give diagnostic criteria, if applicable
Data sources/ measurement 105-126 133-150	8*	For each variable of interest, give sources of data and details of methods of assessment (measurement). Describe comparability of assessment methods if there is more than one group
Bias 130	9	Describe any efforts to address potential sources of bias
Study size 99-102	10	Explain how the study size was arrived at
Quantitative variables 158-160, 165-167	11	Explain how quantitative variables were handled in the analyses. If applicable, describe which groupings were chosen and why
Statistical methods 152-167	12	(*a*) Describe all statistical methods, including those used to control for confounding
NA		(*b*) Describe any methods used to examine subgroups and interactions
99-102		(*c*) Explain how missing data were addressed
149-150		(*d*) If applicable, explain how loss to follow-up was addressed
NA		(*e*) Describe any sensitivity analyses
**Results**		
Participants 99-102	13*	(a) Report numbers of individuals at each stage of study—eg numbers potentially eligible, examined for eligibility, confirmed eligible, included in the study, completing follow-up, and analysed
		(b) Give reasons for non-participation at each stage
		(c) Consider use of a flow diagram
Descriptive data 170-177, Table 1	14*	(a) Give characteristics of study participants (eg demographic, clinical, social) and information on exposures and potential confounders
99-102		(b) Indicate number of participants with missing data for each variable of interest
189		(c) Summarise follow-up time (eg, average and total amount)
Outcome data 189-190	15*	Report numbers of outcome events or summary measures over time

Main results 193-236 Tables 1-3	16	(*a*) Give unadjusted estimates and, if applicable, confounder-adjusted estimates and their precision (eg, 95% confidence interval). Make clear which confounders were adjusted for and why they were included
		(*b*) Report category boundaries when continuous variables were categorized
		(*c*) If relevant, consider translating estimates of relative risk into absolute risk for a meaningful time period
Other analyses NA	17	Report other analyses done—eg analyses of subgroups and interactions, and sensitivity analyses
**Discussion**		
Key results 239-242	18	Summarise key results with reference to study objectives
Limitations 262-269	19	Discuss limitations of the study, taking into account sources of potential bias or imprecision. Discuss both direction and magnitude of any potential bias
Interpretation 242-274	20	Give a cautious overall interpretation of results considering objectives, limitations, multiplicity of analyses, results from similar studies, and other relevant evidence
Generalisability 262-263	21	Discuss the generalisability (external validity) of the study results
**Other information**		
Funding Funding page	22	Give the source of funding and the role of the funders for the present study and, if applicable, for the original study on which the present article is based

*Give information separately for exposed and unexposed groups.

Note: An Explanation and Elaboration article discusses each checklist item and gives methodological background and published examples of transparent reporting. The STROBE checklist is best used in conjunction with this article (freely available on the Web sites of PLoS Medicine at http://www.plosmedicine.org/, Annals of Internal Medicine at http://www.annals.org/, and Epidemiology at http://www.epidem.com/). Information on the STROBE Initiative is available at http://www.strobe-statement.org.
